# Ultraviolet‐Resistant Flexible Perovskite Solar Cells with Enhanced Efficiency Through Attachable Nanophotonic Downshifting and Light Trapping

**DOI:** 10.1002/smll.202501374

**Published:** 2025-03-03

**Authors:** Jae‐Won Kim, Suji Kim, Na‐Kyung Lee, Ha‐Eun Cho, Seung Jun Park, Jae‐Hyun Kim, Nohyun Lee, Sun‐Kyung Kim, Seok Ho Cho, Sung‐Min Lee

**Affiliations:** ^1^ Department of Electrical Engineering Hanyang University Seoul 04763 Republic of Korea; ^2^ Interdisciplinary Program for Photonic Engineering Chonnam National University Gwangju 61186 Republic of Korea; ^3^ School of Materials Science and Engineering Kookmin University Seoul 02707 Republic of Korea; ^4^ School of Electrical Engineering Korea Advanced Institute of Science and Technology Daejeon 34141 Republic of Korea; ^5^ Department of Applied Physics Kyung Hee University Yongin 17104 Republic of Korea; ^6^ Department of Clothing and Textiles Chonnam National University Gwangju 61186 Republic of Korea; ^7^ Division of Materials Science and Engineering Hanyang University Seoul 04763 Republic of Korea

**Keywords:** downshifting, flexible solar cells, nanostructured photonic sticker, perovskite solar cells, ultraviolet stability

## Abstract

Despite the many promising properties of perovskite solar cells (PSCs), ultraviolet (UV)‐induced degradation remains a critical issue for their long‐term reliability. One potential solution is the selective inhibition of UV exposure before it reaches the PSCs; however, this approach leads to a reduction in PSC efficiency due to limited photon utilization. In this regard, here a universally applicable method is presented to address the UV stability issue of PSCs without compromising their high‐level efficiency while also providing device flexibility. A UV‐absorbing colorless polyimide (CPI) substrate serves as a flexible protective shield against UV illumination. The photocurrent loss in CPI‐based PSCs is mitigated by a nanostructured photonic sticker that incorporates a UV‐to‐visible downshifting medium, which can be easily integrated with the fabricated PSC substrate. Through the combined effects of downshifting and synergistic light trapping, the efficiency of UV‐resistant CPI‐based PSCs is improved from 18.6% to 20.4%, making it comparable to the performance of UV‐damageable glass‐based PSCs. Together with numerical modeling, various experimental characterizations of optical and photovoltaic properties, as well as stability assessments under UV, bending, and off‐normal incidence conditions, provide insights into the underlying physical phenomena and optimal design considerations for successful application.

## Graphical abstract

A broadly applicable strategy to achieve UV resistance, device flexibility, and uncompromised photovoltaic performance in perovskite solar cells is presented using a colorless polyimide with abrupt UV cut‐off behavior and a nanostructured transparent luminescent sticker incorporating Eu‐complex photon downshifter. Through the combined effects of downshifting and synergistic light trapping, the efficiency of UV‐resistant PSCs is increased to 20.4%.

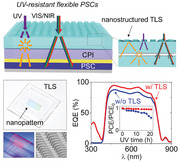

## 1 Introduction

Perovskite solar cells (PSCs) are emerging as promising renewable energy solutions due to their advantageous features, including remarkable efficiency, simple fabrication processes, and flexible module designs.^[^ ^1^, ^2^, ^3^, ^4^ ^]^ The high absorption coefficient (≈10^5^ cm^−1^) and long exciton diffusion length (≈1 mm) of perovskite active layers contribute to their relatively superior power conversion efficiency (PCE) compared to other types of solar cells,^[^ ^5^, ^6^, ^7^ ^]^ positioning PSCs prominently in the photovoltaic society. Recent developments have demonstrated highly efficient PSCs achieving PCE values surpassing 26%,^[^ ^8^ ^]^ placing them in substantial parity with state‐of‐the‐art solar cell technologies. However, PSCs face challenges related to limited stability under operating conditions, including joule/radiation heating, oxygen/moisture permeation, and ultraviolet (UV) exposure,^[^ ^9^, ^10^, ^11^, ^12^, ^13^, ^14^, ^15^ ^]^ which hinders their commercial viability. These challenges have triggered extensive research focused not only on improving PCE but also on addressing the instability of PSCs.

Technical advancements aimed at enhancing PSC stability by preventing thermal degradation and oxygen/moisture‐induced phase decomposition involve the incorporation of inorganic materials and the implementation of solid encapsulation systems.^[^ ^16^, ^17^, ^18^, ^19^, ^20^, ^21^ ^]^ Meanwhile, to address the stability issue under UV exposure, the introduction of UV‐blocking filters on the incident plane or the modification of perovskite interface materials has been suggested.^[^ ^22^, ^23^, ^24^, ^25^, ^26^, ^27^ ^]^ Both methods are effective; however, the former is more straightforward and preferable due to its universal applicability, regardless of cell structure. Despite being conceptually ideal, the UV filter technology has a significant drawback: the limited photon utilization for photocurrent generation compromises the PCE of PSCs. In this context, employing downshifting materials can be an effective option to alleviate the PCE decrease while still providing a UV‐blocking function, as UV solar photons are converted into lower‐energy photons before reaching the photoactive perovskite materials.^[^ ^28^, ^29^, ^30^, ^31^ ^]^ Nevertheless, typical downshifting systems cannot serve as a complete solution for several reasons. First, incomplete cut‐off behavior, arising from the spectrally non‐abrupt absorption band, leads to either incomplete UV photon inhibition (i.e., inferior stability improvement) or reduced visible photon incidence on PSCs (i.e., PCE degradation).^[^ ^22^, ^32^, ^33^, ^34^, ^35^ ^]^ Second, the considerable loss of downshifted photons due to their isotropic emission nature limits the effectiveness of photon retrieval, resulting in minor PCE improvement.^[^ ^36^, ^37^, ^38^ ^]^

In this study, we present fully UV‐resistant flexible PSCs with enhanced PCE by utilizing a UV‐absorbing colorless polyimide (CPI) substrate and an easily combinable nanostructured downshifting medium. The aromatic CPI, known for thermal stability and chemical resistance, acts as a UV filter owing to its complete opacity in the UV range.^[^ ^23^, ^39^, ^40^, ^41^ ^]^ The nanostructured downshifting medium, referred to here as a nanostructured transparent luminescent sticker (TLS), enables UV‐to‐visible photon conversion and light trapping, thereby enhancing the photocurrent of PSCs. The key features of the nanostructured TLS include i) reducing photon escape at the TLS/air interface for efficient guiding of downshifted photons to PSCs, ii) diffracting and anti‐reflecting incident solar photons to enhance photon absorption in PSCs, and iii) simple attachment as a sticker to pre‐existing CPI‐based PSCs, minimizing integration complexity. Systematic investigations based on both experimental characterization and numerical modeling provide a comprehensive understanding of the underlying physical phenomena and optimal design considerations, demonstrating successful high‐performance UV‐resistant flexible PSCs.

## 2 Results and Discussion

### 2.1 Device Design and Working Principle

**Figure 1:**
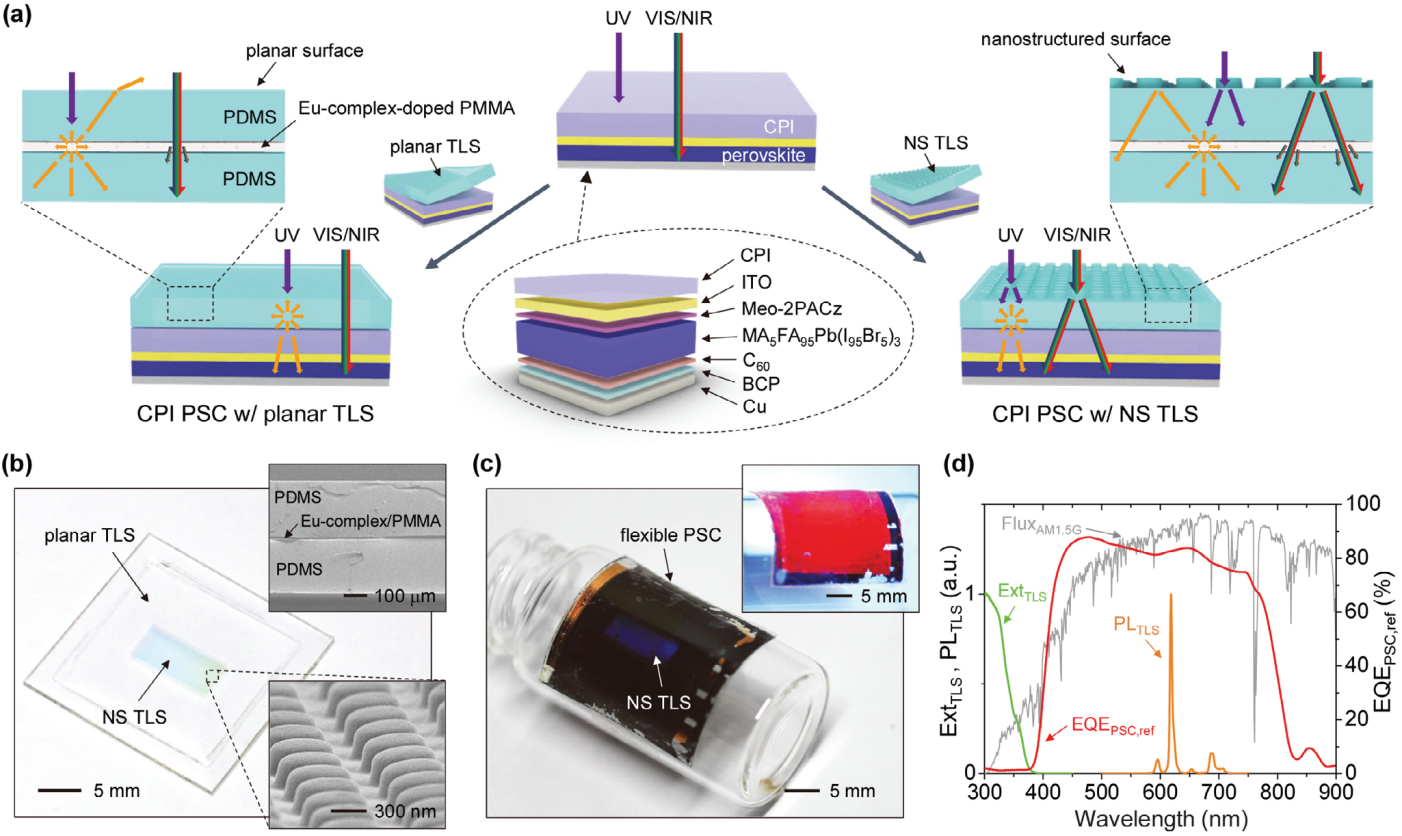
Working principle of UV‐resistant flexible PSCs with TLS. a) Schematic illustrations of the device design and its optical operation process under the solar spectrum. The left and right schematics indicate CPI‐based PSCs with planar TLS and nanostructured (NS) TLS, respectively. b) Photographic image of the fabricated TLS sample under ambient light. The upper and lower insets show scanning electron microscope (SEM) images of the TLS cross‐section and the tilted‐view TLS nanostructure surface, respectively. c) Photographic images of bent PSCs with TLS under ambient and UV (inset) light. d) Measured extinction (Ext_TLS_) and photoluminescence (PL_TLS_, under excitation of 315 nm) spectra of TLS, along with measured EQE spectrum of the control CPI‐based PSC without TLS (EQE_PSC,ref_). The photon flux spectrum of AM1.5G (Flux_AM1.5G_, unit: 3 × 10^17^ s^−1^ m^−2^ nm^−1^, left *y*‐axis) is also displayed for comparison.

**Figure** **1**a schematically illustrates the device design and working principle. We employed a type of high‐performance single‐junction inverted PSC consisting of an anode of indium tin oxide (ITO, 150 nm), a hole transport layer of (2‐(3,6‐dimethoxy‐9H‐carbazol‐9‐yl)ethyl)phosphonic acid (MeO‐2PACz, 10 nm), a perovskite photoactive layer of methylammonium formamidinium lead iodide bromide (MA_5_FA_95_Pb(I_95_Br_5_)_3_, ≈650 nm), an electron transport layer of fullerene C60 (C_60_, 23 nm), a hole blocking layer of bathocuproine (BCP, 8 nm), and a cathode of copper (Cu, 100 nm).^[^ ^42^ ^]^ On a CPI substrate (50 µm), this PSC exhibits a PCE of ≈18.6% with a short‐circuit current density (*J*_sc_) of ≈21.0 mA cm^−2^, an open‐circuit voltage (*V*_oc_) of ≈1.08 V, and a fill factor (FF) of ≈82.0%, showing minor hysteresis behavior (Figure S1, Supporting Information). Note that the PSC with an identical layer structure on a glass substrate demonstrates a PCE of ≈20.4% (*J*_sc_ = ≈23.5 mA cm^−2^, *V*_oc_ = ≈1.09 V, FF = ≈79.9%). The attachable TLS consists of polydimethylsiloxane (PDMS), europium (Eu)‐complex‐doped poly(methyl methacrylate) (PMMA), and another PDMS (thickness of PDMS/PMMA/PDMS = ≈240/3/240 µm), with Eu‐complex luminophores doped in the PMMA interlayer at a weight fraction (*f*_Eu_) ranging from 5.4 to 18.6 wt%. The planar or nanostructured TLS is attached to the CPI substrate of the PSC without adhesive, owing to the self‐adhesive property of the flat PDMS surface.^[^ ^43^, ^44^ ^]^ For the nanostructured TLS, a rectangular array of nanoposts with near‐optimal dimensions (period; *p* = 900 nm, diameter; *D* = 600 nm, height; *h* = 350 nm) is formed on one outer surface of the TLS PDMS through a simple molding process (see the fabrication procedure in Figure S2, Supporting Information). Figure 1b and Figure S3 (Supporting Information) show the fabricated nanostructured TLS, where uniformly formed nanopattern and its resulting diffraction color can be identified. The integrated configuration of nanostructured TLS with PSCs is depicted in Figure 1c, demonstrating the flexible device configuration and effective working under UV light.

Since the 50 µm‐thick CPI substrate completely blocks UV light with wavelengths below 385 nm (Figure S4, Supporting Information), the external quantum efficiency (EQE) of the CPI‐based PSCs is nearly zero at these wavelengths. The Eu‐complex of TLS absorbs UV photons exclusively within this zero‐level EQE range of the CPI‐based PSCs and emits visible photons at ≈618 nm (Figure 1d) through the Stokes shift process, thus enabling the CPI‐based PSC to utilize UV photons without any detrimental effects on the existing photovoltaic performance. To attain the maximum benefit from the attached TLS, isotropically emitted Eu‐complex photons should be efficiently delivered to the PSC. As described in Figure 1a, the planar TLS is disadvantaged in photon delivery to PSCs, as it loses a significant quantity of downshifted photons due to emission inside an air escape cone (angle = ≈43.6°) or being waveguided to the edge of the slab. By contrast, the nanostructure at the outer surface of the TLS can suppress photon loss even inside an air escape cone and diffract waveguided photons toward the opposite PSC direction, concentrating more photons on the PSC. Furthermore, the nanostructured TLS surface exhibits diffraction of the incident visible and near‐infrared (NIR) solar photons, which extends the photon travel length. Synergistically combined with the photon scattering behavior of the Eu‐complex luminophores, this diffraction property of the TLS nanostructure substantially contributes to photon absorption in PSCs.

### 2.2 Numerical Study for TLS Nanostructures

**Figure 2:**
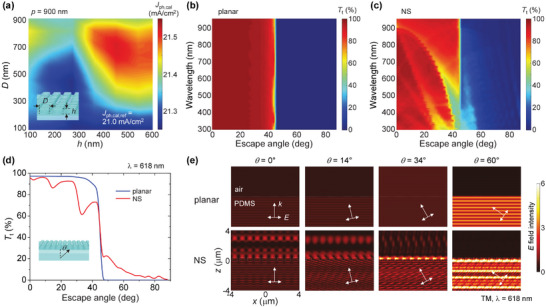
Numerical optical calculations for TLS nanostructures. a) Contour plot of the calculated *J*_ph_ (*J*_ph,cal_) for the CPI‐based PSC with Eu‐complex‐free TLS as a function of *D* and *h* of rectangular‐arranged nanoposts on the TLS PDMS surface, with *p* = 900 nm. The *J*_ph,cal_ value for the control device without TLS (*J*_ph,cal,ref_) is also provided for comparison. b,c) Contour plots of the calculated *T*_t_ from PDMS to air for planar (b) and nanostructured (NS, *p*/*D*/*h* = 900/600/350 nm) (c) TLS PDMS surfaces at various wavelengths and escape (incident) angles. d) Calculated *T*_t_ spectra for planar and nanostructured TLS PDMS surfaces at the Eu‐complex emission wavelength of 618 nm. e) Contour plots of the electric (*E*) field distribution for planar and nanostructured TLS PDMS surfaces when TM‐polarized 618 nm plane waves propagate from PDMS to air at representative incident angles of 0°, 14°, 34°, and 60°.

The photonic resonance at the nanostructure interface can modulate the field density profile, resulting in a denser diffracted field on the side with a higher refractive index. This modulation causes an anti‐reflection effect when photons move from a lower‐refractive‐index medium to a higher‐refractive‐index medium and an anti‐transmission effect when photons move in the opposite direction.^[^ ^45^, ^46^ ^]^ Translating this behavior to the present system, it can be understood as follows: Due to the outer surface nanostructure of the TLS PDMS, incident visible solar photons can move to the PSC more effectively (anti‐reflection), while emitted photons from the TLS PMMA can be preferentially guided to the PSC (anti‐transmission). To quantitatively assess these effects of the TLS PDMS nanostructure, we conducted numerical simulation modeling using the finite‐difference time‐domain (FDTD) method, as illustrated in **Figure** **2**. First, we determined the optimal dimensions of the nanopost array to maximize the anti‐reflection effect for incident solar photons. Figure 2a shows the calculated photocurrent density, denoted as *J*_ph,cal_ for the calculated *J*_ph_, at various values of *D* and *h* of nanoposts under a fixed rectangular period of 900 nm. *J*_ph,cal_ was derived from an equation based on detailed balance analyses,^[^ ^47^, ^48^, ^49^, ^50^ ^]^ given by,

(1) $$J_{\mathrm{ph},\mathrm{cal}}=q\int_{0}^{\infty }\mathrm{EQE}\left(E\right)\Phi_{T}\left(E\right)dE$$

where *q*, EQE, Φ_T_, and *E* represent the electron charge, the experimental EQE of the control CPI‐based PSCs (i.e., without the TLS), the transmitted solar flux at the air/PDMS interface, and the photon energy, respectively. Although the air/PDMS interface has a low refractive index contrast (*Δn* = *n*_PDMS_–*n*_air_ = 0.4), which results in relatively low photonic resonance intensity, a noticeable enhancement in *J*_ph,cal_ is observed at specific dimensions (*D* > ≈300 nm, *h* > ≈350 nm). This suggests that the nanostructured PDMS itself can meaningfully augment the photocurrent of PSCs.

The anti‐transmission effect alleviating photon escape was tested using experimental near‐optimal nanopost dimensions (*p* = 900 nm, *D* = 600 nm, *h* = 350 nm). Figure 2b,c depict the total (i.e., collimated and diffuse) transmission (*T*_t_) from PDMS to air for various escape angles and wavelengths. In this analysis, oblique incidence cases with transverse magnetic (TM) or transverse electric (TE) polarizations were calculated separately (Figure S5, Supporting Information) and then averaged in consideration of the random polarization in Eu‐complex photon emission. For the planar PDMS surface (Figure 2b), propagating photons with angles below a critical angle of 45.6° mostly escape from the PDMS to air, causing significant photon loss. By contrast, for the nanostructured PDMS surface (Figure 2c), substantial photons traveling within the escape cone cannot be transmitted if resonance conditions are satisfied, leading to reduced photon escape loss. Figure 2d provides the *T*_t_ spectra for the planar and nanostructured PDMS surfaces at the Eu‐complex emission wavelength of 618 nm. Transmission dips are observed at the nanostructured surface case for escape angles of ≈14° and ≈34°, indicating that the resonances generated under these angle conditions provoke a reduction in the photon escape loss. While photons traveling outside the escape cone (θ > 45.6°) partially exit to the air at the nanostructured surface due to leaky mode coupling,^[^ ^51^, ^52^ ^]^ their quantity is relatively small. The electric field distributions for TM‐polarized plane waves at selected escape angles (θ = 0°, 14°, 34°, 60°) are shown in Figure 2e, where distinct resonance behaviors are identified in the nanostructured surface case.

### 2.3 Optical Properties of Experimental TLSs

**Figure 3:**
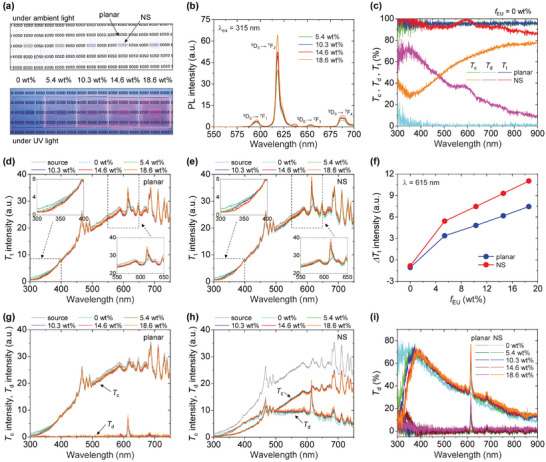
Optical characterizations of experimental TLSs. a) Photographic images of TLS samples (25 × 25 mm^2^) with planar and nanostructured (NS, *p*/*D*/*h* = 900/600/350 nm) surfaces under ambient and UV light, with *f*_Eu_ values of 0, 5.4, 10.3, 14.6 and 18.6 wt%. b) PL intensity spectra for nanostructured TLS with various *f*_Eu_ values under 315 nm excitation. The state transitions are also indicated. c) *T*_c_, *T*_d_, and *T*_t_ spectra for planar and nanostructured TLSs without Eu‐complex (*f*_Eu_ = 0 wt%). d,e) *T*_t_ intensity spectra for planar (d) and nanostructured (e) TLSs with various *f*_Eu_ values. The inset graphs manifest selected ranges magnified for clarity. f) Change in *T*_t_ intensity at the emission wavelength of 615 nm for planar and nanostructured TLSs as a function of *f*_Eu_, relative to the source intensity. g,h) *T*_c_ intensity and *T*_d_ intensity spectra for planar (g) and nanostructured (h) TLSs with various *f*_Eu_ values. i) *T*_d_ spectra for planar and nanostructured TLSs with various *f*_Eu_ values.

To characterize the fabricated TLSs before integration with the PSC, we implemented representative optical measurements at various sample conditions. **Figure** **3**a displays photographic images of the TLS samples with both planar and nanostructured surfaces under ambient or UV light. It is evident that the planar TLS area is fully transparent, whereas the nanostructured TLS area appears transparent but exhibits haziness. Under UV light, photon emission from the Eu‐complex is identified, where the stronger emission is observed at the TLS sample with a higher *f*_EU_. The nanostructure pattern area is distinct under the UV light in all *f*_EU_ cases, indicating effective diffraction of Eu‐complex photons. The corresponding observations can be found in the photoluminescence (PL) intensity spectra in Figure 3b, measured at 60° against the surface normal under an excitation wavelength (λ_ex_) of 315 nm. A strong emission peak (≈618 nm) of the ^5^D_0_ to ^7^F_2_ transition is monitored in the Eu‐complex‐doped TLS,^[^ ^32^, ^53^, ^54^ ^]^ where all emission peaks are intensified if *f*_EU_ increases.

The transmission measurements provide insights into the anti‐transmission effect for Eu‐complex photons, as well as the diffraction and scattering effects for incident solar photons. For these measurements, a xenon lamp served as a light source, and a fiber‐optic Fourier‐transform spectrometer recorded the spectrum. The transmission levels were calculated by normalizing the transmission intensity of the sample against the source intensity. First, we compared the collimated (*T*_c_), diffuse (*T*_d_), and total (*T*_t_) transmissions of the TLS samples without the Eu‐complex luminophores (i.e., no downshifting) to verify the diffraction of incident photons by the TLS nanostructure. As shown in Figure 3c, the *T*_t_ of the nanostructured TLS is comparable (>90%) to that of the planar TLS across all wavelengths, occasionally approaching 100% (i.e., the anti‐reflection effect) at wavelengths ≈600 nm, where high solar photon flux is present. In contrast to the negligible *T*_d_ of the planar TLS, substantial *T*_d_ values are observed in the nanostructured TLS due to surface diffraction. Figure 3d,e show the *T*_t_ intensity spectra of planar and nanostructured TLS samples, respectively. Since incident photons with *λ* < 380 nm are absorbed and converted by the Eu‐complex inside the TLS, the *T*_t_ intensity gradually decreases at these wavelengths and exceeds the source intensity at the emission wavelengths (e.g., *λ*_em_ = ≈618 nm) as *f*_EU_ increases. The analogous reductions in *T*_t_ intensity at *λ* < 380 nm for both planar and nanostructured TLSs imply that similar photon quantities are involved in the downshifting process for each TLS. However, the *T*_t_ intensity of the nanostructured TLS is significantly higher at the emission wavelength compared to the planar TLS, as highlighted by the peak value comparison at *λ* = ≈615 nm in Figure 3f. This validates that the downshifted Eu‐complex photons are more effectively directed toward the back side (i.e., the anti‐transmission effect by the TLS nanostructure surface). As depicted in Figure 3g,h, the generation of Eu‐complex photons is monitored in the *T*_d_ intensity spectra but not in the *T*_c_ intensity spectra, due to the isotropic emission nature. Notably, overall values of the *T*_d_ intensity for the nanostructured TLS rise slightly with increasing *f*_EU_, whereas those for the planar TLS remain consistently close to zero. This wavelength‐independent increase in *T*_d_ with *f*_EU_ demonstrates the photon scattering behavior by Eu‐complex luminophores, which becomes evident under off‐normal photon propagation (e.g., diffracted photon condition). Although only the *T*_d_ spectra are provided in Figure 3i to highlight the scattering effect, the corresponding *T*_c_ and *T*_t_ spectra for all samples can be found in Figure S6 (Supporting Information) for comparison.

### 2.4 Photovoltaic Characterization of Integrated PSCs

**Figure 4:**
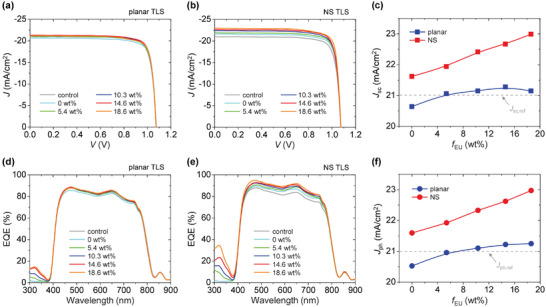
Photovoltaic performance of experimental CPI‐based PSCs integrated with TLS. a,b) Representative *J*–*V* curves for the devices with planar (a) and nanostructured (b) TLSs at various *f*_Eu_ values, measured under the AM1.5G solar spectrum. The control device without TLS is also shown for comparison. c) Measured *J*_sc_ values for the devices with planar and nanostructured TLSs as a function of *f*_Eu_. d,e) Measured EQE spectra for the devices with planar (d) and nanostructured (e) TLSs corresponding to (a) and (b). f) Derived *J*_ph_ values from the EQE spectra under AM1.5G for the devices with planar and nanostructured TLSs as a function of *f*_Eu_.

**Figure** **4**a,b show the current density (*J*)‐voltage (*V*) curves of the CPI‐based PSCs with planar or nanostructured TLS, respectively, measured under the simulated AM1.5G standard solar spectrum (100 mW cm^−2^) at room temperature. The derived photovoltaic parameters, given in **Table** **1**, manifest that the addition of TLS primarily affects the optical parameter *J*_sc_ rather than the electrical parameters *V*_oc_ and FF. Note that an identical PSC sample was employed for all TLS cases in these measurements to avoid potential influences from sample‐to‐sample variation, thereby strictly isolating the effect of TLS integration. The variation in *J*_sc_ of the TLS‐integrated PSCs with respect to changes in *f*_EU_ is summarized in Figure 4c. At *f*_EU_ = 0 wt% (i.e., no Eu‐complex), *J*_sc_ for the planar TLS case is slightly lower compared to the control PSC (*ΔJ*_sc_ = −0.37 mA cm^−2^) because of photon reflection loss within the TLS (PDMS/PMMA/PDMS); in contrast, increased *J*_sc_ is observed for the nanostructured TLS case (*ΔJ*_sc_ = 0.61 mA cm^−2^) from diffraction‐induced photon absorption enhancement. While the planar TLS case presents a minor gain in *J*_sc_ as *f*_EU_ increases, the nanostructured TLS case shows a notable improvement. As discussed earlier, this is due to the preferential guidance of downshifted Eu‐complex photons to the PSC (i.e., enhanced UV photon utilization) and the synergistic scattering of diffracted solar photons (i.e., boosted visible/NIR photon trapping). By integrating the nanostructured TLS, the CPI‐based PSC can advance its PCE from 18.6% (before integration) up to 20.4%. This represents that the UV‐resistant flexible CPI‐based PSC can achieve a high PCE level comparable to that of the UV‐damageable rigid glass‐based PSC (Figure S7, Supporting Information).

**Table 1:** Photovoltaic characteristics of CPI‐based PSCs with various TLS configurations under the AM1.5G solar spectrum.

	*J*_sc_ [mA cm^−2^]	*V*_oc_ [V]	FF [%]	PCE [%]
**Control**	21.01^a)^ (20.84 ± 0.15)^b)^	1.081 (1.081 ± 0.001)	82.0 (80.9 ± 0.7)	18.63 (18.24 ± 0.17)
planar TLS, 0 wt%	20.64 (20.48 ± 0.12)	1.079 (1.082 ± 0.002)	81.9 (80.5 ± 0.9)	18.24 (17.85 ± 0.21)
planar TLS, 5.4 wt%	21.06 (20.88 ± 0.12)	1.081 (1.083 ± 0.002)	82.1 (80.7 ± 1.0)	18.69 (18.26 ± 0.21)
planar TLS, 10.3 wt%	21.15 (20.97 ± 0.13)	1.079 (1.082 ± 0.002)	82.7 (81.4 ± 0.7)	18.88 (18.46 ± 0.18)
planar TLS, 14.6 wt%	21.28 (21.10 ± 0.12)	1.080 (1.084 ± 0.003)	82.7 (81.2 ± 0.9)	19.02 (18.59 ± 0.22)
planar TLS, 18.6 wt%	21.15 (21.01 ± 0.13)	1.079 (1.084 ± 0.002)	82.5 (81.2 ± 0.9)	18.82 (18.49 ± 0.19)
NS TLS, 0 wt%	21.62 (21.45 ± 0.12)	1.082 (1.084 ± 0.001)	82.5 (81.8 ± 0.7)	19.29 (19.03 ± 0.15)
NS TLS, 5.4 wt%	21.94 (21.74 ± 0.13)	1.081 (1.083 ± 0.003)	82.7 (81.9 ± 0.5)	19.63 (19.29 ± 0.16)
NS TLS, 10.3 wt%	22.42 (22.22 ± 0.11)	1.081 (1.083 ± 0.002)	82.0 (81.7 ± 0.5)	19.87 (19.68 ± 0.12)
NS TLS, 14.6 wt%	22.67 (22.48 ± 0.14)	1.081 (1.084 ± 0.002)	82.7 (82.0 ± 0.8)	20.26 (19.97 ± 0.14)
NS TLS, 18.6 wt%	22.99 (22.81 ± 0.13)	1.080 (1.084 ± 0.002)	82.3 (82.0 ± 0.6)	20.44 (20.28 ± 0.09)

^a)^ These are the values attained from a representative PSC;

^b)^ These values indicate the average and standard deviation obtained from 8 different PSCs, each with identical TLS where applicable.

The EQE response measurements in Figure 4d,e support the earlier descriptions of *J*_sc_ enhancement in the CPI‐based PSC through TLS integration. Since the CPI substrate blocks photons with *λ* < 380 nm, the apparent EQE values in this UV wavelength range, occurring only for the TLS‐integrated PSCs, indicate photocurrent generation from downshifted photon absorption in the PSC. These UV‐range EQE values are significantly higher for the nanostructured TLS case than for the planar TLS case at identical *f*_EU_, meaning that the downshifted photons are more effectively absorbed in the PSC with the nanostructured TLS. Compared to the planar TLS case, the nanostructured TLS case exhibits a considerable increase in EQE across the entire visible and NIR ranges, as well as in the UV range, with increasing *f*_EU_. This increase in the visible/NIR‐range EQE is a consequence of the photon scattering effect of Eu‐complex luminophores, as recognized in the transmission measurements. Figure 4f provides the *J*_ph_s attained from the measured EQE responses under the assumption of the AM1.5G solar spectrum, where consistency between *J*_ph_ and *J*_sc_ for the identical device case is identified. In the meantime, if intensified UV is used as an illumination source, such TLS integration can substantially boost the photocurrent of CPI‐based PSCs compared to standard solar illumination conditions (Figure S8, Supporting Information).

### 2.5 Reliability Tests of Integrated PSCs

**Figure 5:**
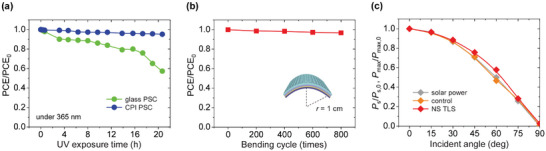
Experimental tests for stability and off‐normal operation. a) Measured PCE/PCE_0_ variations for glass‐based and CPI‐based PSCs exposed to intensified UV (100 W, 365 nm) as a function of exposure time. b) Measured PCE/PCE_0_ variation for CPI‐based PSC with nanostructured TLS as a function of bending deformation cycle (radius, *r* = 1 cm). c) Calculated incident solar power ratio (*P*_s_/*P*_s,0_ = cos θ) and measured *P*_max_/*P*_max,0_ for CPI‐based PSCs with and without nanostructured TLS at different incident angles. PCE_0_ in (a) and (b) and *P*_max,0_ in (c) represent the initial values under the AM1.5G solar spectrum.

Due to the complete UV‐filtering behavior of the CPI substrate, the proposed PSC device is effectively protected from UV‐induced degradation. **Figure** **5**a and Figure S9 (Supporting Information) present a comparison of the measured photovoltaic performance variations for the glass‐based PSC and the TLS‐integrated CPI‐based PSC, both stored under intensified UV exposure (a 365 nm‐centered UV source with 100 W power) in an ambient environment (room temperature, 40% relative humidity). The PCE of the UV‐permeable glass‐based PSC decreases by over 40% of its initial value after 20 h of UV exposure. By contrast, the PCE of the CPI‐based PSC remains almost constant after the same exposure time (less than a 5% reduction of the initial value), validating the expected UV resistance.

The mechanically flexible operation is another compelling property of the CPI‐based PSC integrated with the conformable TLS. To demonstrate performance stability under repeated deformations, we monitored the PCE variation with respect to the bending iteration at a fixed bending radius of 1 cm. As shown in Figure 5b, the PCE remained nearly consistent after 800 bending cycles (≈3% reduction of the initial value), indicating reasonable mechanical stability. Meanwhile, the TLS integration contributes to the PSC performance more successfully under oblique photon incidence due to the increased downshifting and scattering events, which result from the extended photon travel within the Eu‐complex luminophore medium. This effect is illustrated by measurements of the maximum output power (*P*_max_) variation for the CPI‐based PSCs with and without the nanostructured TLS at various incident angles of the solar spectrum (Figure 5c). As the incident angle increases, the *P*_max_ of the TLS‐free PSC drops at a rate consistent with the diminishing projection power of the solar illumination; in contrast, that of the TLS‐integrated PSC decreases more slowly. The TLS effectiveness under off‐normal illumination is particularly beneficial for flexible‐mode operation, as not all of the photovoltaic area is exposed to normal illumination.

## 3 Conclusion

In summary, we suggested a broadly applicable strategy to achieve UV resistance, device flexibility, and uncompromised photovoltaic performance in PSCs by utilizing a CPI substrate with abrupt UV cut‐off behavior and a nanostructured TLS incorporating the Eu‐complex UV‐to‐visible photon converter. The nanostructured TLS reduced photon escape loss for downshifted photons and effectively trapped incident visible/NIR solar photons through photonic diffraction and synergistic photon scattering, resulting in enhanced photocurrent. The integration of the nanostructured TLS improved the PCE of the CPI‐based PSCs from 18.6% to 20.4% while maintaining device stability under UV exposure and repeated deformation.

This study demonstrated the nanostructured TLS to enhance the performance of flexible PSCs formed on a UV‐filter plastic substrate. Since the TLS and UV‐filter substrate are external elements that do not directly affect the electrical properties of the PSCs, the proposed strategy is valid in principle for various material combinations in PSCs, including heat‐resistant materials, beyond the employed design. While the effectiveness of the nanostructured TLS was elucidated for UV‐filtered flexible PSCs, the reported concept can also be applied to UV‐permeable PSCs that can benefit from photon energy modulation and light trapping for performance enhancement, regardless of device flexibility, though the UV stability issue may not be fully resolved. We anticipate that the present strategy will offer a versatile solution for addressing the UV stability issue in various PSCs without compromising photovoltaic performance.

## 4 Experimental Section

### Fabrication of Nanostructured TLSs

The fabrication process for the TLS began with the preparation of PMMA solutions containing Eu‐complex luminophores, which were synthesized using methods reported in the literature.^[^ ^32^ ^]^ Bathophenanthroline (2 mmol, Sigma–Aldrich) and europium nitrate hexahydrate (1 mmol, Alfa Aesar) were each separately dissolved in 20 mL of ethanol. These two solutions were then mixed together and stirred for 12 h. The resulting mixture was centrifuged at 3000 rpm for 10 min and washed with ethanol. After three cycles of centrifugation and washing, the solution was dried at 60 °C in a convection oven for 12 h, yielding purified Eu‐complex powder. This powder was then dispersed in a dichloromethane (Sigma–Aldrich) solution containing PMMA (70 mg mL^−1^), with the amount of Eu‐complex powder adjusted to prepare solutions with varying *f*_EU_ levels (5.4, 10.3, 14.6, 18.6 wt%). Next, a fluorinated delamination polymer (Novec 1700) was spin‐coated onto cleaned glass and annealed at 190 °C for 15 min. The prepared PMMA solution with Eu‐complex was then spin‐coated (1000 rpm, 30 s) onto the polymer‐coated glass and baked at room temperature. On this Eu‐complex‐doped PMMA film, PDMS with a base‐to‐curing agent ratio of 12:1 was spin‐coated at 500 rpm for 30 s and cured at 50 °C for 12 h. Subsequently, the Eu‐complex‐doped PMMA/PDMS layers were peeled off and were ready to be combined with another PDMS layer that had a nanostructure. This additional PDMS layer was separately prepared by spin‐coating PDMS (base/curing agent = 10:1 by weight) on a KrF‐lithographically patterned nanohole mold (*p*/*D*/*h* = 900/600/350 nm) at 500 rpm for 30 s, followed by curing at 50 °C for 12 h. After the nanostructured PDMS was peeled off, its flat side was gently attached to the previously prepared Eu‐complex‐doped PMMA/PDMS layers. Schematic descriptions of the fabrication process appear in Figure S2 (Supporting Information).

### Fabrication of CPI‐Based PSCs

On a cleaned 50 µm‐thick CPI substrate (25 × 25 mm^2^, SH Sigma), patterned indium tin oxide (ITO, 150 nm) was deposited using a sputter system. After UV‐ozone treatment for 30 min, MeO‐2PACz (Tokyo Chemical Industry) dissolved in anhydrous ethanol (1 mmol/L) was spin‐coated at 3000 rpm for 30 s and annealed at 100 °C for 10 min in an atmospheric environment. In a nitrogen‐filled glove box, a perovskite precursor solution was then spin‐coated at 4000 rpm for 35 s, while a chlorobenzene anti‐solvent was simultaneously dropped onto the perovskite layer 5 s before the end of the spin‐coating process. The sample was then annealed at 100 °C for 60 min. The perovskite precursor solution used at this step was prepared by the following process. First, stock solutions were made by dissolving lead iodide (PbI_2_, Tokyo Chemical Industry) and lead bromide (PbBr_2_, Tokyo Chemical Industry) in a mixed solution of anhydrous dimethylformamide (DMF) and dimethyl sulfoxide (DMSO) (4:1 by volume) at a nominal concentration of 1.5 m. Formamidinium iodide (FAI, Greatcell Solar Materials) and methylammonium bromide (MABr, Greatcell Solar Materials) were then added to the PbI_2_ and PbBr_2_ stock solutions, respectively, to obtain FAPbI_3_ and MAPbBr_3_ solutions at 1.24 m (with a lead‐to‐cation ratio of 1.09:1). These FAPbI_3_ and MAPbBr_3_ solutions were mixed in a 95:5 volume ratio to form the MA_5_FA_95_Pb(I_95_Br_5_)_3_ solution, followed by the addition of methylammonium chloride (MACl, Greatcell Solar Materials) dissolved in DMSO (5:95 by volume) to complete the perovskite precursor solution. Meanwhile, as a final step in PSC fabrication, C_60_ (23 nm, Nano‐C), BCP (8 nm, Tokyo Chemical Industry), and Cu (100 nm) were sequentially deposited on the MA_5_FA_95_Pb(I_95_Br_5_)_3_ perovskite‐coated sample using a thermal evaporator system with a shadow mask.

### Measurements of Optical and Photovoltaic Properties

The PL intensities of the TLSs were assessed using a spectrofluorophotometer (F‐7000, Hitachi) at the excitation wavelength of 315 nm. The transmission intensities of the TLSs were monitored with a spectrometer (Maya 2000 Pro, Ocean Optics) equipped with an integrating sphere (CSRM‐RTC‐060‐SL, Labsphere). A high‐power xenon lamp (1000 W, Newport) was used as a light source. The photovoltaic characteristics of the PSCs were analyzed using a Class AAA full‐spectrum solar simulator (K3000, McScience) with a xenon arc lamp, calibrated with a certified silicon reference cell. The *J*‐*V* curves were scanned using a source meter (Series 2400, Keithley), with a high scan rate of 250 mV s^−1^ to enable clear observation of potential hysteresis during the scan process.^[^ ^55^ ^]^ The illuminated cell area was defined by a shadow mask to 2 × 2 mm^2^. The EQE spectra of the PSCs were collected using a commercial quantum efficiency measurement system (K3100 EQX, McScience), calibrated with a silicon reference cell. The monochromatic light was obtained by spectroscopic separation from a white light source (xenon lamp, 300 W).

### Numerical Optical Simulation

Optical transmission of PDMS nanostructures was simulated by the FDTD method (Lumerical FDTD, Ansys). For the normal incidence case, a 3D simulation volume was defined with periodic boundary conditions in the x‐/y‐directions and a ʻperfectly matched layers (PML)ʼ boundary condition in the z‐direction. A light source of continuous plane‐wave with a broad Gaussian frequency spectrum (315–999 THz) was used. For the oblique incidence case, a ʻBlochʼ boundary condition was applied in the x‐/y‐directions, along with a continuous plane‐wave characterized by a narrow Gaussian frequency spectrum centered at a desired wavelength. The refractive index of PDMS was set to 1.4 based on the literature.^[^ ^56^ ^]^

### Statistical Analysis

To compare the photovoltaic performances of PSCs with various TLS configurations, a representative PSC was used, and different TLSs were subsequently applied. The trend of performance variation observed from the representative PSC was confirmed by testing 8 different PSCs with the same TLSs. Both the photovoltaic parameters obtained from the representative PSC and their average and standard deviation values from the 8 different PSCs are provided in Table 1.

## Conflict of Interest

The authors declare no conflict of interest.

## Supporting information



Supporting Information

## Data Availability

The data that support the findings of this study are available from the corresponding author upon reasonable request.
