# Supplementary material for: Ultraviolet‐Resistant Flexible Perovskite Solar Cells with Enhanced Efficiency Through Attachable Nanophotonic Downshifting and Light Trapping
Source: Small. 2025 Mar 3;21(24):2501374. doi: 10.1002/smll.202501374 (PMC12177856; doi:10.1002/smll.202501374)
Supplement: Supplementary file 1 — Supporting Information [file SMLL-21-2501374-s001.pdf]

NANO · MICRO

small

Supporting Information

for *Small*, DOI 10.1002/smll.202501374

Ultraviolet-Resistant Flexible Perovskite Solar Cells with Enhanced Efficiency Through Attachable Nanophotonic Downshifting and Light Trapping

Jae-Won Kim, Suji Kim, Na-Kyung Lee, Ha-Eun Cho, Seung Jun Park, Jae-Hyun Kim, Nohyun Lee, Sun-Kyung Kim, Seok Ho Cho and Sung-Min Lee**

Copyright WILEY-VCH Verlag GmbH & Co. KGaA, 69469 Weinheim, Germany, 2025.

Supporting Information

Ultraviolet-Resistant Flexible Perovskite Solar Cells with Enhanced Efficiency through Attachable Nanophotonic Downshifting and Light Trapping

Jae-Won Kim,[+] Suji Kim,[+] Na-Kyung Lee, Ha-Eun Cho, Seung Jun Park, Jae-Hyun Kim, Nohyun Lee, Sun-Kyung Kim, Seok Ho Cho,[*] Sung-Min Lee[*]

Table of Contents

Supporting Figures and Captions

Figure S1 ~ S9

Supporting Figures and Captions

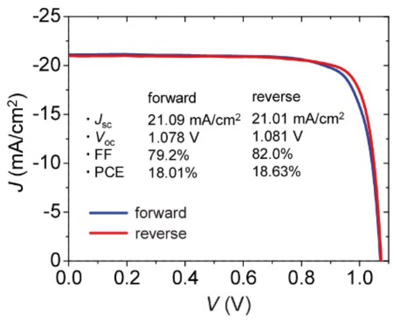

Figure S1. Hysteresis behavior of experimental CPI-based PSCs. Representative *J-V* curves scanned during forward and reverse voltage sweeps, measured under the AM1.5G solar spectrum. The derived photovoltaic parameters from these measurements are also provided.

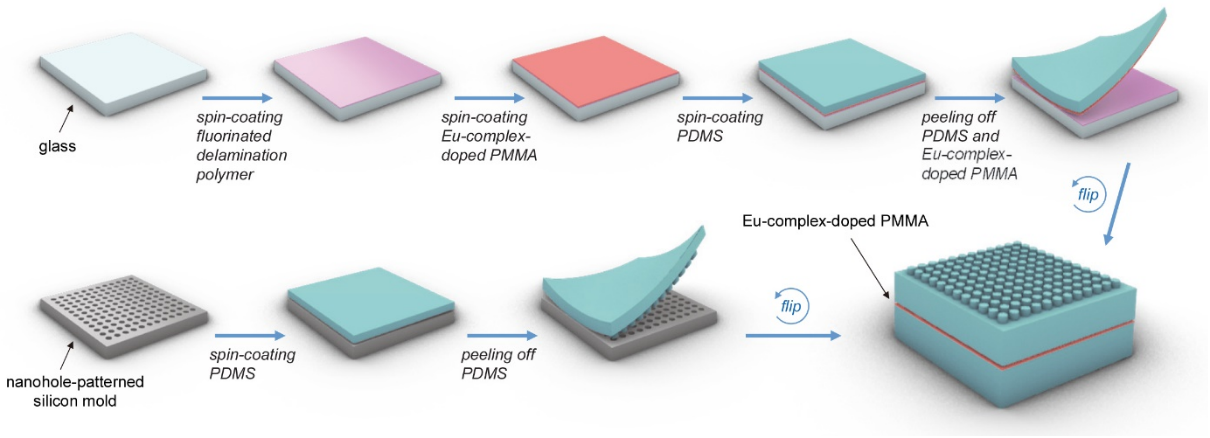

Figure S2. Fabrication of nanostructured TLSs. Schematic illustration of the process steps involved in fabricating the nanostructured TLS.

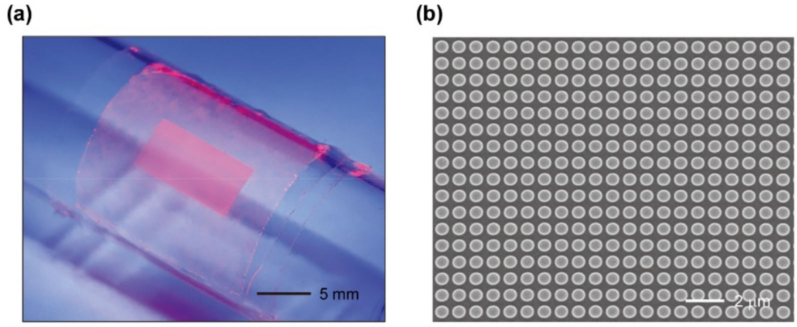

Figure S3. Experimental TLS samples. (**a**) Photographic image of the fabricated TLS in the bent state under UV light. (**b**) Top-view SEM image of the TLS nanostructure surface (nanopost: $p/D/h$ = 900/600/350 nm).

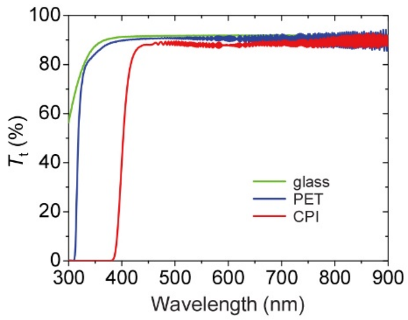

Figure S4. Optical measurements of representative substrates for PSCs. Measured T_t spectra for glass (thickness; t = 700 μm), polyethylene terephthalate (PET) (t = 30 μm), and CPI (t = 50 μm) substrates.

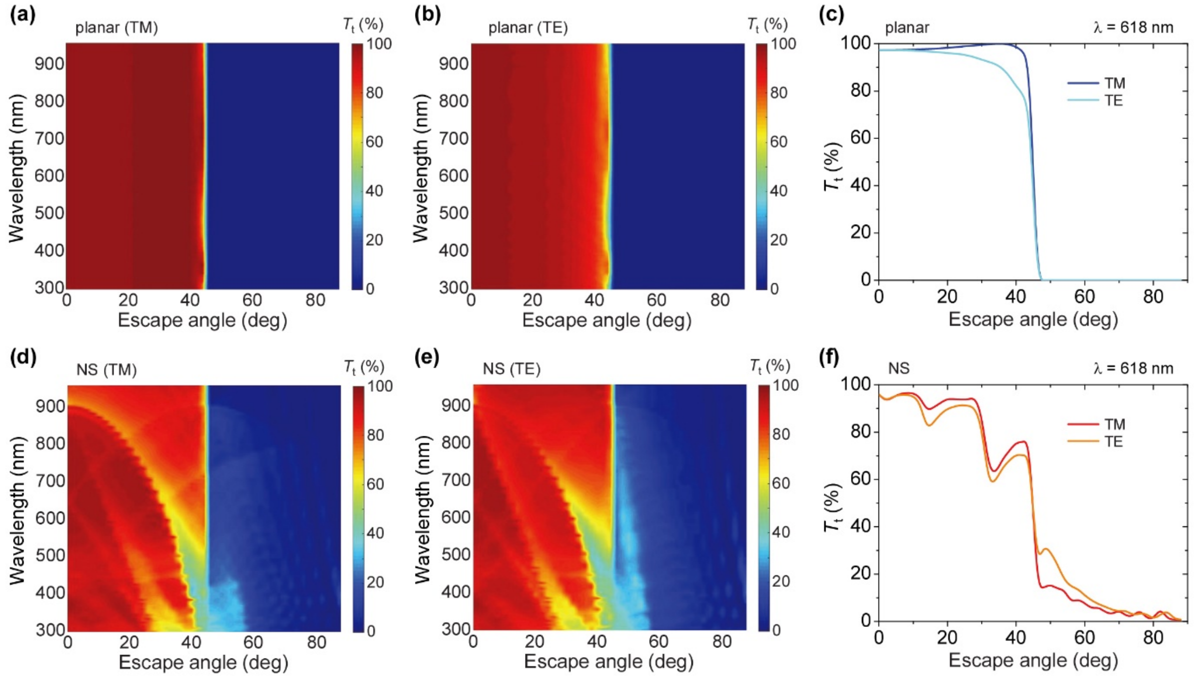

Figure S5. Optical simulations for planar and nanostructured TLS surfaces. (**a**,**b**) Contour plots of TM-polarized (**a**) and TE-polarized (**b**) total transmission (T_t) from PDMS to air for the planar TLS PDMS surface at various wavelengths and escape (incident) angles. (**c**) T_t spectra for TM-polarized and TE-polarized light at the Eu-complex emission wavelength of 618 nm for the planar TLS PDMS surface. (**d**), (**e**), and (**f**) correspond to (**a**), (**b**), and (**c**), respectively, but for the nanostructured (NS, *p*/*D*/*h* = 900/600/350 nm) TLS PDMS surface.

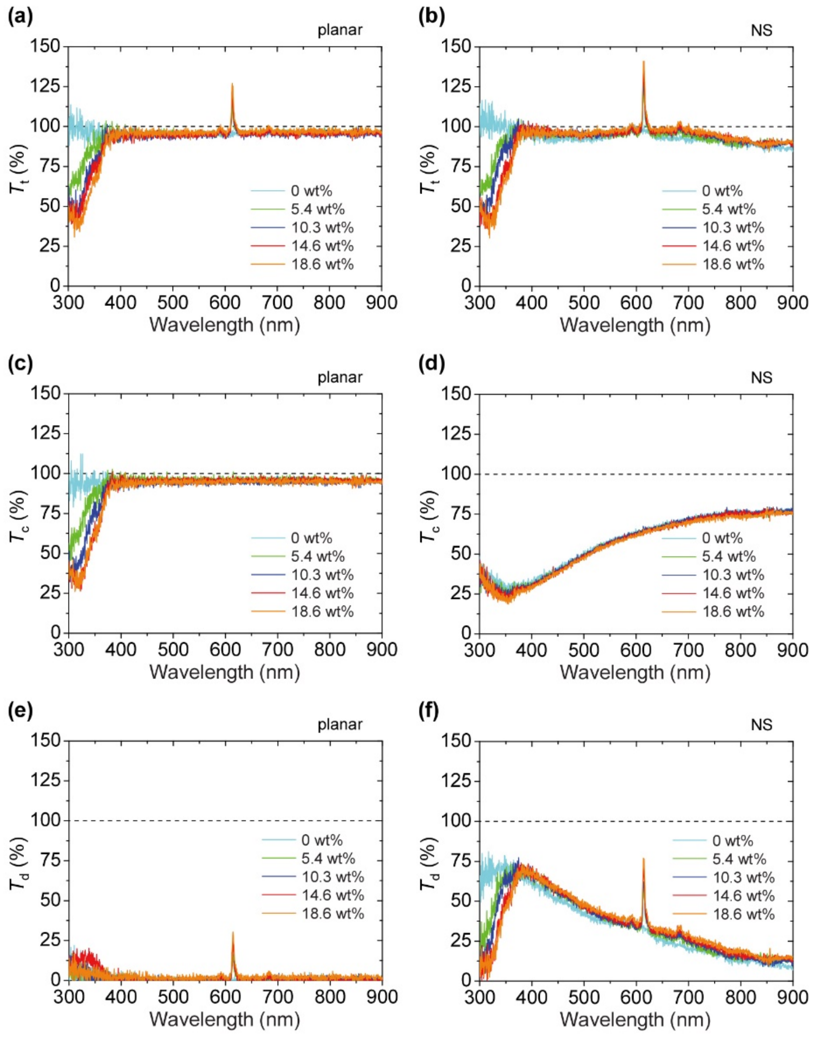

Figure S6. Optical measurements of experimental TLSs. (**a**,**b**) Measured T_t spectra for planar (**a**) and nanostructured (NS, *p*/*D*/*h* = 900/600/350 nm) (**b**) TLSs with various f_{Eu} values. (**c**,**d**) Measured T_c spectra for TLSs corresponding to (**a**) and (**b**), respectively. (**e**,**f**) Measured T_d spectra for TLSs corresponding to (**a**) and (**b**), respectively.

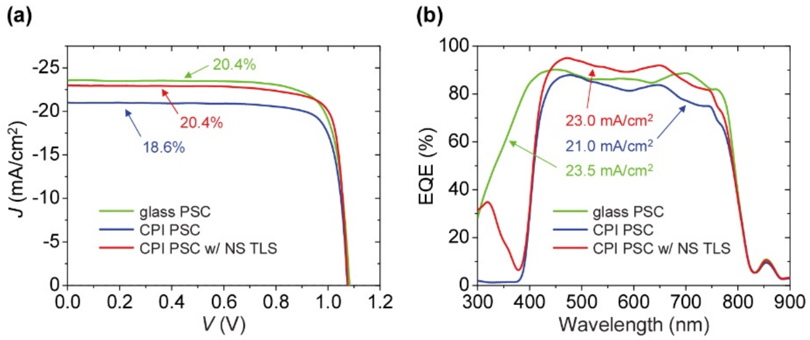

Figure S7. Comparison of photovoltaic performances for glass-based and CPI-based PSCs. (**a**) Representative *J-V* curves for glass-based PSC, CPI-based PSC, and CPI-based PSC with nanostructured ($p/D/h$ = 900/600/350 nm) TLS, measured under the AM1.5G solar spectrum. (**b**) Measured EQE spectra for the devices corresponding to (**a**), along with the derived J_{ph} values under AM1.5G.

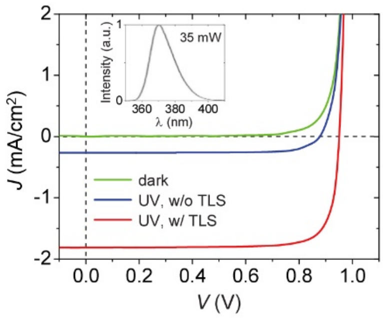

Figure S8. Photovoltaic characteristics of CPI-based PSCs under intensified UV light. *J-V* curves for the devices with and without nanostructured ($p/D/h$ = 900/600/350 nm) TLS under a 35 mW UV light source with spectral response depicted in the inset. The dark-state *J-V* curve of the device is also provided.

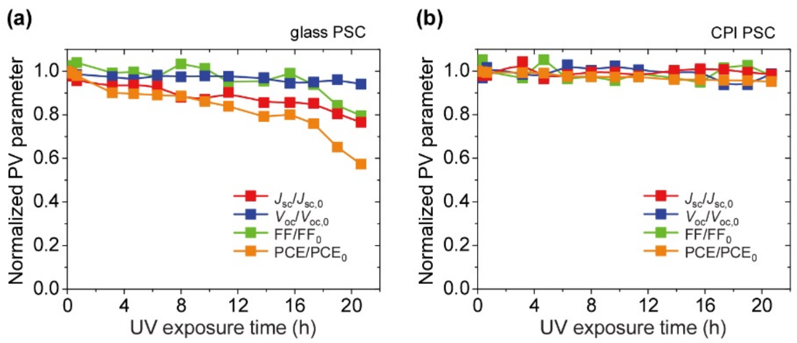

Figure S9. Performance variation of PSCs during UV exposure. Measured $J_{sc}/J_{sc,0}$, $V_{oc}/V_{oc,0}$, FF/FF$_0$, and PCE/PCE$_0$ for glass-based (**a**) and CPI-based (**b**) PSCs exposed to intensified UV (100 W, 365 nm) as a function of exposure time. $J_{sc,0}$, $V_{oc,0}$, FF$_0$, and PCE$_0$ indicate the initial values of the photovoltaic parameters under the AM1.5G solar spectrum.
